# Clinical Perspectives of Urocortin and Related Agents for the Treatment of Cardiovascular Disease

**DOI:** 10.1155/2012/198628

**Published:** 2012-04-03

**Authors:** Keiichi Ikeda, Kouki Fujioka, Yoshinobu Manome, Katsuyoshi Tojo

**Affiliations:** ^1^Department of Molecular Cell Biology, Institute of DNA Medicine, Research Center for Medical Sciences, Jikei University School of Medicine, Tokyo 105-8461, Japan; ^2^Division of Diabetes and Endocrinology, Department of Internal Medicine, Jikei University School of Medicine, Tokyo 105-8461, Japan

## Abstract

The effects of corticotropin-releasing hormone, also known as corticotropin-releasing factor (CRF), on the cardiovascular system have been intensively researched since its discovery. Moreover, the actions of urocortin (Ucn) I on the cardiovascular system have also been intensively scrutinized following the cloning and identification of its receptor, CRF receptor type 2 (CRFR2), in peripheral tissues including the heart. Given the cardioprotective actions of CRFR2 ligands, the clinical potential of not only Ucn I but also Ucn II and III, which were later identified as more specific ligands for CRFR2, has received considerable attention from researchers. In addition, recent work has indicated that CRF type 1 receptor may be also involved in cardioprotection against ischemic/reperfusion injury. Here we provide a historical overview of research on Ucn I and related agents, their effects on the cardiovascular system, and the clinical potential of the use of such agents to treat cardiovascular diseases.

## 1. Introduction: Overview of Corticotropin-Releasing Hormone and Related Peptides and Their Effects on the Cardiovascular System

Since the discovery of a 41-residue ovine hypothalamic peptide that stimulates secretion of corticotropin, several studies have revealed that corticotropin-releasing factor (CRF) affects the cardiovascular system, although the CRF receptors involved in this process had not been identified [1–9]. In 1995, a major breakthrough in research on the effects of CRF-related peptides on the cardiovascular system took place with the cloning and identification of the CRF type 2 receptor (CRFR2) in peripheral tissues including the cardiovascular system [10–14]. This finding indicated that CRF and related peptides may affect the heart and vascular tissues. In addition, urocortin (Ucn) I, which was later recognized as a key CRF peptide in the heart, was also identified in rat mid brain tissue [15], while Ucn I mRNA, but not CRF, was identified in cardiomyocytes [16]. Several studies revealed that stimulation of CRFR2 by CRF and Ucn I induced the release of atrial and brain natriuretic peptides (ANP and BNP, resp.) [17, 18], which are used as the indicators of cardiac hypertrophy [19] and, in another hand, have an antihypertrophic action [20], had a positive inotropic action on the heart [21], increased protein and DNA synthesis in cardiac fibroblasts [18, 22, 23], and exerted a cardioprotective action against hypoxia [16, 24, 25]. Ucn I immunoreactivity has also been identified in normal and diseased human heart, especially in the hearts of patients with dilated and hypertrophic cardiomyopathies (DCM and HCM, resp.) [22, 26, 27]. The other reported beneficial actions of Ucn I on the heart is to reduce infarct size in vivo, improve intracellular calcium handling [28], increase the ventricular fibrillation threshold [29], reduce the occurrence of arrhythmias [28], and inhibit efferent cardiac sympathetic nerve activity [30]. These facts indicated that Ucn I and its analogs may have beneficial actions in the treatment of cardiac diseases as well as play certain roles in cardiac diseases. Furthermore, CRFR2, which is a potential receptor of Ucns in the heart, may play an important role in adaptation to cardiac stress [31], and dominant negative effects of a recently identified variant isoform of CRFR2 may play a critical role in the pathophysiology of stress-induced heart disease [32]. These findings indicate that CRFR2 signaling is important in the adaptation to cardiac stress and heart-related diseases. In addition to Ucn I, the Ucn I analogs Ucn II and Ucn III, which are more specific ligands of CRFR2, were successively identified in searches of publicly available human genome databases [33, 34]. Ucn II and Ucn III were also identified in human and rodent heart [35–37]. Thereafter, more studies on the effects of Ucn I and related peptides were undertaken.

CRFR2 was also identified in the aorta-derived A7R5 cell line [38], and Ucn I was identified in human umbilical vein endothelial cells (HUVECs) [39]. Ucn I and its analogs exert vasodilatory effects in arteries and veins via their action on CRFR2 [40–42]. Furthermore, Ucn I exerts an antioxidative action in response to the angiotensin II-induced generation of reactive oxygen species (ROS) by HUVECs [39]. In other studies, however, Ucn I was shown to exert proinflammatory effects by augmenting via CRFR2 the lipopolysaccharide-induced expression of cyclooxygenase (COX)-2 and intercellular adhesion molecule-1 in rat aortic endothelial cells and to induce vasculitis via CRF type 1 receptor (CRFR1) [43, 44].

## 2. CRFR1 and CRFR2 Signaling in the Cardiovascular System and Cardioprotective Action of Ucns

Several studies have described the signal transduction pathway for CRFR2. Recently, the CRFR1 was identified in HL-1 cardiomyocytes in addition to CRFR2, and the possible involvement of CRFR1-mediated extracellularly regulated kinase1/2 (ERK1/2) signaling pathways in Ucns' cardioprotective action against ischemia/reperfusion injury was indicated [45]. It has been suggested that the CRFR1 and CRFR2 signal transduction pathways in cardiovascular cells involve protein kinase A (PKA) [18, 35, 40], Src [45], p38 mitogen-activated kinases (MAPKs) [40, 46], ERK1/2 [45, 47–49], the protein kinase C (PKC) pathway [50, 51], the protein kinase B/Akt pathway [36, 48, 49], phosphoinositide-3 kinase (PI3-K) [49], the COX-2 pathway [52], and the endothelial nitric oxide synthase pathway [48]. For example, the hypertrophy-inducing action of Ucns, which is manifested by an increase of ANP and BNP secretion from cardiomyocytes and an increase in protein synthesis, may be induced via the PKA and Akt pathways [18, 35, 36], while the cardioprotective action of Ucns against hypoxia and reperfusion injury may involve the Src, ERK1/2, PKC, and PI3-K pathways [45, 49, 51].

## 3. Clinical Use of Ucns to Treat Cardiovascular Disease

Some clinical applications of Ucns in cardiovascular diseases, such as ischemic heart disease, cardiac failure, and hypertension, among others, have been proposed.

### 3.1. Ischemic Heart Disease

The relationship between the action of CRFR2 and ischemia-induced cardiomyocyte damage has been extensively investigated [16, 24, 25, 36, 53]. Ucn I clearly exerts an anti-ischemic action on cardiomyocytes. The protective action of Ucns against ischemia could be mediated via CRFR2 and, in turn, via the ERK1/2, MAPK, and PI3-K pathways [24, 53]. In addition, pretreatment with Ucn I to protect against ischemia resulted in the significant recovery of high-energy phosphate pools [25]. The recent study suggested the expression of CRFR1 and possible involvement of the CRFR1 signaling pathway in cardioprotection against ischemic/reperfusion injury [45]. These findings indicate that Ucns and related agents (i.e., CRFR1 and CRFR2 agonists) could serve as candidate therapeutic agents to combat ischemic heart diseases.

### 3.2. Heart Failure

Elevated immunoreactivity to Ucn I in diseased heart was identified in DCM [22]. Our group also detected increased Ucn I immunoreactivity in the heart of DCM and HCM patients [27]. Furthermore, several studies reported that plasma Ucn I immunoreactivity was elevated in patients with cardiac failure and ischemic heart disease and in sheep with experimental cardiac failure [54–58]. Elevated plasma Ucn I concentrations combined with N-terminal proBNP may enhance prognostic performance in acute myocardial infarction [56]. On the other hand, several trials in which Ucns are being used to treat experimental [59–64] and human cardiac failure [65] are ongoing and have provided some potentially beneficial results, because Ucn I may exert inotropic actions that may play a key role in the treatment of cardiac failure. And Ucn II may also improve the renal function [66], and sympathetic activity in heart failure [30], synthetic Ucns, or nonpeptide CRF receptor agonists may prove useful for the treatment of cardiac failure.

### 3.3. Hypertension

One of the actions of Ucns on the vasculature is to cause vasodilation. In addition to these vasodilatory actions, Yang et al. suggested that Ucn I may decrease the activity of angiotensin-converting enzyme and, in turn, reduce blood pressure [67, 68]. To this extent, infusion of Ucn II in healthy humans and in patients with cardiac failure resulted in a decrease of blood pressure [65, 69]. However, clinical data on the treatment of hypertension with Ucns are not yet available.

In conclusion, Ucn-related agents (synthetic CRF receptor agonists) may have clinical potential for the treatment of patients with clinically manifested cardiovascular diseases. Accumulating evidence of the beneficial actions of Ucns will serve as the experimental basis for the clinical use of Ucn-related compounds.
